# Measurement of Enteric Methane Emissions by the SF_6_ Technique Is Not Affected by Ambient Weather Conditions

**DOI:** 10.3390/ani11020528

**Published:** 2021-02-18

**Authors:** Peter J. Moate, Jennie E. Pryce, Leah C. Marett, Josie B. Garner, Matthew H. Deighton, Brigid E. Ribaux, Murray C. Hannah, William J. Wales, S. Richard O. Williams

**Affiliations:** 1 Agriculture Victoria Research, Ellinbank, Victoria 3821, Australia; leah.marett@agriculture.vic.gov.au (L.C.M.); josie.garner@agriculture.vic.gov.au (J.B.G.); brigid.ribaux@agriculture.vic.gov.au (B.E.R.); murray.hannah@agriculture.vic.gov.au (M.C.H.); bill.wales@agriculture.vic.gov.au (W.J.W.); richard.williams@agriculture.vic.gov.au (S.R.O.W.); 2 Centre for Agricultural Innovation, Faculty of Veterinary and Agricultural Sciences, School of Agriculture and Food, The University of Melbourne, Victoria 3010, Australia; 3 Agriculture Victoria Research, La Trobe University, Bundoora, Victoria 3083, Australia; jennie.pryce@agriculture.vic.gov.au; 4 Cropmark Seeds Ltd., Christchurch 7677, New Zealand; matthew.deighton@cropmark.co.nz

**Keywords:** wind speed, temperature, humidity, rainfall, methane yield, cattle

## Abstract

**Simple Summary:**

Although the SF_6_ technique was developed over 25 years ago with the intention that it could be used to measure enteric methane production from ruminants outdoors, no experiments have reported the influence of ambient wind speed, temperature, humidity or rainfall on the accuracy of the technique. Six different cohorts of dairy cows (40 per cohort) were kept outdoors and fed a common diet during spring in 3 consecutive years. Individual cow feed intakes and daily methane productions were measured over 5 consecutive days and an automatic weather station measured air temperature, wind speed, relative humidity and rainfall every 10 min. Regression analyses were used to relate the average daily temperature, wind speed, humidity and rainfall to the average daily dry matter intake, methane production and methane yield of each cohort of cows. It was concluded that the modified SF_6_ technique can be used outdoors during a range of weather conditions without a significant effect on the measurement of methane production or methane yield of dairy cows.

**Abstract:**

Despite the fact that the sulphur hexafluoride (SF_6_) tracer technique was developed over 25 years ago to measure methane production from grazing and non-housed animals, no studies have specifically investigated whether ambient wind speed, temperature, relative humidity and rainfall influence the accuracy of the method. The aim of this research was to investigate how these weather factors influence the measurement of enteric methane production by the SF_6_ technique. Six different cohorts of dairy cows (40 per cohort) were kept outdoors and fed a common diet during spring in 3 consecutive years. Methane production from individual cows was measured daily over the last 5 days of each 32-day period. An automated weather station measured air temperature, wind speed, relative humidity and rainfall every 10 min. Regression analyses were used to relate the average daily wind speed, average daily temperature, average daily relative humidity and total daily rainfall measurements to dry matter intake, average daily methane production and methane yield of each cohort of cows. It was concluded that the modified SF_6_ technique can be used outdoors during a range of wind speeds, ambient temperatures, relative humidities and rainfall conditions without causing a significant effect on the measurement of methane production or methane yield of dairy cows.

## 1. Introduction

There are five main methods used to measure daily methane production (MeP, g/day) and methane yield (MeY, g/kg dry matter intake). The respiration chamber method is considered the “gold standard” [1] and has been used by many researchers over the past 5 decades. Two recently developed methods include the sniffer method [2] and the Greenfeed or C-lock method [3] which have been increasingly used in recent research [4]. However, due to the limited sampling times used in these latter methods [5], and the marked diurnal patterns in the magnitude of MeP from dairy cows [6], some researchers have questioned the suitability of these recent methods for quantifying daily MeP of individual cows [7,8]. Proxy methods for estimating daily MeP or MeY of individual animals are numerous, including predictions based on dry matter intake (DMI) [9], feed composition [10], methane to carbon dioxide ratio in breath [11], milk fatty acids [12], and volatile fatty acids in ruminal fluid [13]. However, proxy methods have generally had poor predictive accuracy and therefore have limited applicability in terms of predicting MeP from individual animals [14]. Lastly, the sulphur hexafluoride (SF_6_) tracer method that was developed by Zimmerman [15] and first used in nutrition research by Johnson et al. [16], has subsequently been used in numerous experiments conducted both indoors and outdoors. Indeed, Ulyatt et al. [17] pointed out that “the SF_6_ technique must be the preferred method for animal scientists because it can be used under normal grazing conditions, data can be obtained from individual animals, and it allows the imposition of experimental treatments”.

The SF_6_ technique employs a permeation tube that releases SF_6_ gas into the reticulo-rumen at a known, constant rate. Eructated gases containing both methane and SF_6_ are collected into evacuated canisters, and the ratio of methane to SF_6_ in the eructated gases can be used to estimate daily MeP [18]. However, large variations in results have been reported when the SF_6_ technique was used [19,20]. The incorrect sampling of background gases has been identified as one source of variation [21], as has the decline in gas sampling rate due to type of flow restrictor [22]. Astute choices in the sampling of background gases and flow restrictor have reduced the variation, but variation still exists. Weather conditions could also be one source of the residual variation.

There have been over 250 papers published describing the use of the SF_6_ technique, but the effects of weather on the technique have not been fully investigated. The concentration of collected breath gases have been reported to decrease as wind speed increased [17]. Wind speed is expected to affect breath sampling efficiency since an increase in wind speed will result in a dilution of exhaled air at the sampling point [17]. While the variation in gas collection appeared to be linked with the calculated MeP, DMI was not measured [17] and therefore it is not possible to determine if the effect of wind speed was on DMI with its resulting MeP [9] or if the effect was on the SF_6_ technique itself. 

Temperature and humidity are known to affect the DMI of dairy cows [23,24], with cows eating less when the temperature and humidity are high. Since DMI and MeP are closely linked [9], any temperature or humidity-induced changes in DMI will result in changes in MeP. Temperature and humidity could also affect the collection of gas samples due to variation in the dispersal of the expired gas plume. The density of gas decreases as temperature and humidity increase [25]. Therefore, since expired gases are generally warmer and wetter than ambient background air, they are less dense and will disperse more quickly when the ambient air is cool and dry. As ambient air temperature and humidity increase, the difference between the densities of ambient air and expired gases will decrease, potentially resulting in increases in the concentrations of SF_6_ and methane in collected samples of expired gases. However, we do not expect temperature and humidity to influence the concentration of SF_6_ or methane in the samples of background gas, since the density changes due to temperature and humidity are not expected to be sufficient to cause a noticeable change in the concentrations of SF_6_ or methane.

Rainfall may be a proxy for relative humidity since wet days generally have a high relative humidity [26]. Rainfall has also been reported to be negatively correlated with DMI [27], and given the positive correlation between DMI and MeP [9], rainfall could be expected to be negatively correlated with MeP.

The aim of the research presented here was to describe how natural variations in wind speed, ambient temperature, relative humidity, and rainfall influence daily DMI, the concentrations of SF_6_ and methane in background gases and in cows’ breath as well as MeP and MeY as measured by the modified SF_6_ technique. We hypothesized that estimated daily MeP and MeY would be independent of normal day-to-day variation in average daily wind speed, temperature, relative humidity and rainfall.

## 2. Materials and Methods 

This experiment utilised a subset of data from a large experiment aimed at documenting the variation in MeP from individual cows. This experiment involved data collected from 240 Holstein lactating cows that calved between July and September over each of three consecutive years (2015–2017) with experiments conducted in each of the three years from October to December inclusive. All protocols of the experiment were approved by the DJPR Agricultural Research and Extension Animal Ethics Committee (Approval # 2013-14, 2016-12). There were 3 cohorts in 2015, 1 cohort in 2016 and 2 cohorts in 2017. Each cohort consisted of 40 cows with this number limited by the availability of the SF_6_ methane measurement equipment. 

### 2.1. Cows and Diet

Cows were selected from the main herd at the research farm, based on days in milk (DIM). Cows were excluded if they already had an SF_6_ permeation tube in the reticulo-rumen, or for health concerns such as a recent case of clinical mastitis. At the start of their measurement period cows were 110 ± 19.4 (mean ± standard deviation) DIM with 2.5 ± 1.25 lactations and 539 ± 69.8 kg body weight.

**Figure 1:**
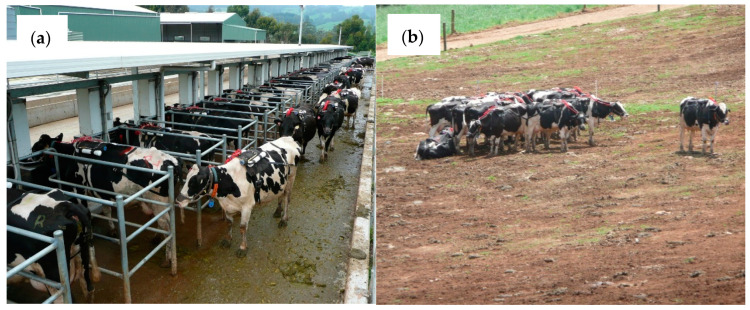
Cows fitted with equipment for methane measurements by the SF_6_ technique and eating outdoors at the auto-recording feed bins (**a**) and cows loafing on a bare paddock (**b**).

For 32 days, each cohort of cows was managed in an experimental facility where they had 24-h/day access to feed and water ad libitum and a bare paddock for rest. Throughout the experiment, cows were outdoors except for twice daily milking events, each of approximately 0.5 h. Individual cow feed intakes were continuously measured by means of feed bins mounted on load cells that were electronically monitored by linking the bin weight data to electronic identification of individual cows (Gallagher Animal Management Systems, Hamilton, New Zealand). The feed bins were located under a small roof to ensure that rain would not compromise the feed intake measurements (see Figure 1a). When not at the feed bins, cows had access to water troughs on a 0.5 ha bare paddock (see Figure 1b). 

The diet consisted of compressed cubes that comprised approximately 74% alfalfa (*Medicago sativa* L.) hay, 25% crushed barley (*Hordeum vulgare* L.) grain, and 1% mineral mix (Multicube Ltd, Yarrawonga, Victoria, Australia). The dimensions of the cubes were approximately 35 × 35 × 55 mm. The alfalfa hay had been finely chopped before cube manufacture and the grain had been ground. 

Individual cow daily DMI (kg/day) were measured over all 32 days. Samples of feed offered and refused were oven dried at 100 °C to a constant weight to determine DM and thus calculate individual DM intake. Representative samples of feed offered and refused were collected daily and pooled per week over the 32-day period (5 samples per cohort of cows). Samples of the cubes offered to cows were oven dried at 60 °C for 24 h, ground to pass through a 0.5 mm screen and then analyzed by the Dairy One Forage Laboratory (Ithaca, NY, USA) for crude protein, soluble crude protein, acid detergent fiber, neutral detergent fiber, lignin, starch, crude fat, ash, calcium, magnesium, phosphorus, potassium, sulphur, and chloride according to their published wet chemistry methods as described by Dairy One [28]. The gross energy was calculated using the approach of Atwater and Woods [29]. 

### 2.2. Measurement of Methane Production

For each cohort of cows, MeP from each individual cow was measured on day 27 to 31 using the modified SF_6_ tracer technique described by Deighton et al. [22]. Briefly, permeation tubes were filled with approximately 2.4 g of SF_6_. The release rate of SF_6_ was 7.2 ± 0.41 mg/day (mean ± standard deviation) and ranged from 6.6 to 8.0 mg/d. The permeation tubes were placed in the reticulo-rumen of the cows per os one week before the first measurements of methane were performed. A canister of 800 mL capacity and a sampling rate of 0.2 mL/min was placed on a saddle on each individual cow and it was used to continuously sample eructated gases from near the mouth of the cow. A second canister that was placed on the saddle of each individual cow was used to sample background gases collected near the paralumbar fossa on the right flank of each cow (see Figure 1a). This system of collecting samples of background gas for each individual cow was employed to take account of the fact that individual cow behavior might influence the concentration of methane and SF_6_ in the background gases to which each cow is exposed. During this experiment, we purposefully kept the cows almost always outdoors (Figure 1b) to ensure relatively low background concentrations of methane and SF_6_, and hence help to optimize conditions necessary for the accurate measurement of methane production by the SF_6_ technique [18]. Canisters were exchanged daily at 07:00 h over the 5-day measurement period. Analysis of collected gas samples was done by gas chromatography [18]. Methane production for each cow on each day were calculated using Equation (2) as presented in the paper of Williams et al. [18].

### 2.3. Weather Data

A weather station (Model J3504; Measurement Engineering Australia, Magill, South Australia, Australia) was located 830 m north-west of the automatic feed facility where the cows were located. During the methane measurement periods in 2015, 2016 and 2017, the weather station measured wind speed, air temperature, and relative humidity every 10 min for the duration of the experiment. These measurements were averaged over each measurement day (07:00 to 06:59) to derive the mean daily temperature (DT, °C), daily wind speed (DW, m/sec) and mean daily humidity (DH, %). Daily mean values were used in preference to spot values, such as maximum temperature, so that weather conditions during gas sampling were reflected in the weather parameters analyzed. Total daily rainfall (DR, mm) was also recorded. These data were available for each of the 5 days of methane measurements for each of the six cohorts resulting in a total of 30 data. 

### 2.4. Calculations and Statistics

The cohort was used as the experimental unit because all cows within a cohort were exposed to the same weather conditions on any given day. Gas data and data on DMI for each cohort of 40 cows were averaged for each day of methane measurement (days 27–31). Resulting variables were average DMI, background concentrations of SF_6_ and methane, concentrations in breath of SF_6_ and methane, MeP, MeY, DW, DT, DH, and DR. 

For each cohort of 40 cows, on each day of gas sampling, data were averaged and plotted against DW, DT, DH and DR. The resulting variables for each day were average DMI (kg/d); average background concentrations of SF_6_ (BGSF6, ppt) and methane (BGCH4, ppm); average concentrations in breath of SF_6_ (CBSF6, ppt) and methane (CBCH4, ppm); average daily MeP; average daily MeY; average daily wind speed (DW, m/sec); average daily temperature (DT, °C), average daily humidity (DH, %) and total daily rainfall (DR, mm).

These cohort by day averaged data were analyzed using a mixed-effects model that included additive linear terms for each of DW, DT, DH and DR as fixed effects, and random terms for cohort and day within cohort. This model was used to test each term using a change-in-deviance F-test, dropping one term out at a time. The R^2^ for each model was calculated using Equation (21) in the paper by Nakagawa and Schielzeth [30].

## 3. Results

The chemical compositions of the feed cubes were similar across all 3 years of observations (Table 1). Mean individual cow DMI ranged from 21.2 to 31.8 kg/day while the cohort mean DMI was 25.3 kg/day.

There was a wide range in the daily weather conditions over the course of our experiment (Table 2). Of the 30 days (six cohorts × 5 days/cohort) during which the SF_6_ technique was used to measure MeP, there were 7 days when maximum daily temperature exceeded 30 °C and there were 16 days when the maximum daily temperature did not exceed 20 °C. There were just three days during which the average daily wind speed was less than 1 m/sec and just 2 days when the average daily wind speed was greater than 3 m/sec.

The SF_6_ technique was applied to a total of 240 cows. A total of 2400 gas samples (1200 individual cow background samples and 1200 individual cow breath samples) were intended to be collected. However, 37 background samples and 51 breath samples were lost due to equipment failure. 

For individual cows, the background concentrations (mean ± standard deviation) of SF_6_ were 17.2 ± 4.12 ppt and for methane 11.2 ± 3.80 ppm, while the concentrations in breath of SF_6_ were 67.3 ± 22.7 ppt and for methane 56.2 ± 17.3 ppm. 

**Figure 2:**
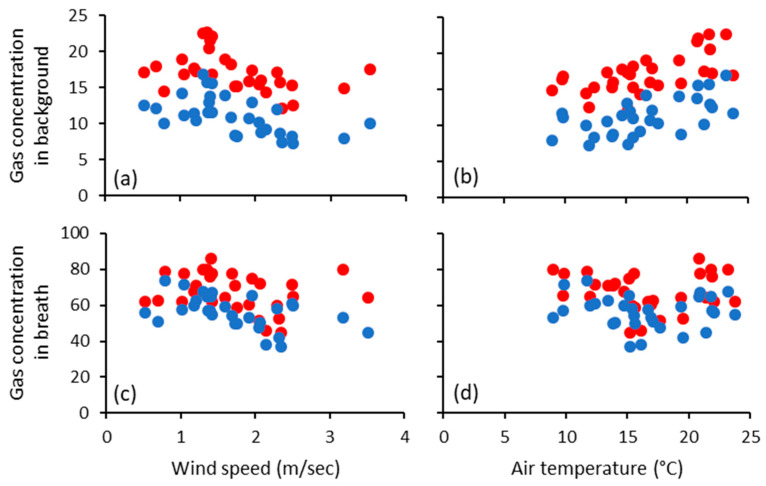
Concentrations of SF6 (●, ppt) and methane (●, ppm) in: background samples as influenced by (**a**) mean daily wind speed, and (**b**) mean daily air temperature; and in breath samples as influenced by (**c**) mean daily wind speed, and (**d**) mean daily air temperature. Note, each datum depicted is the mean of data from 40 individual cows.

The mean daily concentrations of SF_6_ and methane in samples of background gas of the cohorts of cows varied from day to day, with the concentrations being negatively (*p* = 0.001) related to average daily wind speed (Figure 2 and Table 3). Linear relationships between each of the background SF_6_ (*p* < 0.05) and background methane (*p* < 0.05), with air temperature alone as the single independent variable were evident (Figure 2b), but these were not significant after accounting for wind speed, humidity and rainfall (Table 3). The mean daily concentrations of SF_6_ and methane in the breath samples of each cohort were negatively (*p* < 0.01) related to wind speed and negatively (*p* = 0.001) related to air temperature, but positively (*p* < 0.006) related to daily rainfall. Rainfall was not related to mean daily air temperature (*p* = 0.890) nor relative humidity (*p* = 0.600).

**Figure 3:**
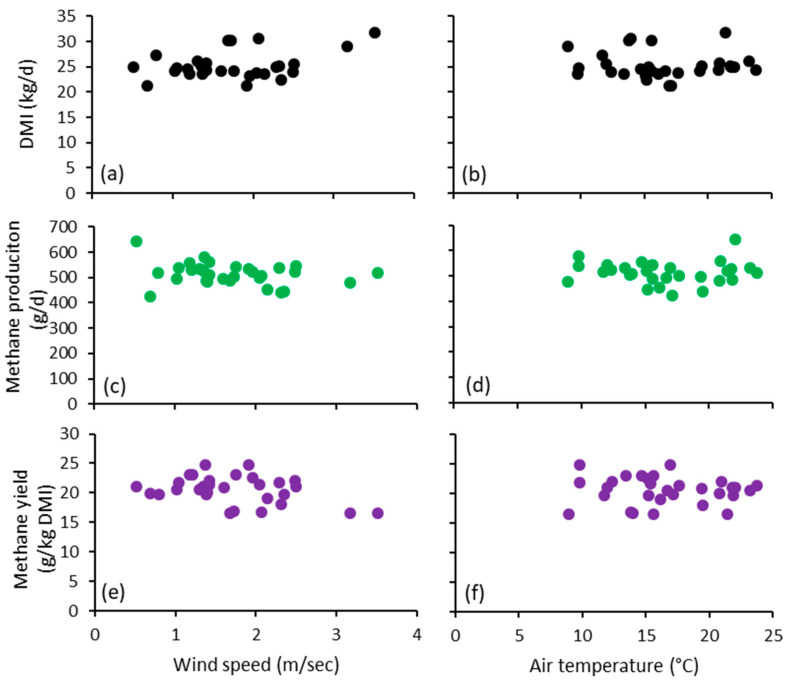
Dry matter intake as influenced by (**a**) mean daily wind speed, and (**b**) mean daily air temperature; methane production (MeP) as influenced by (**c**) mean daily wind speed, and (**d**) mean daily air temperature; methane yield (MeY) as influenced by (**e**) mean daily wind speed, and (**f**) mean daily temperature. Note, each datum depicted is the mean of data from 40 individual cows.

In this experiment, the average daily DMI of cohorts of cows was not related to average daily wind speed (*p* = 0.410) (Table 3 and Figure 3a), average daily temperature (*p* = 0.954) (Table 3 and Figure 3b), average daily humidity (*p* = 0.831) or total daily rainfall (*p* = 0.729). For each cohort of cows, the average daily MeP was not related to average daily wind speed (*p* = 0.072) (Table 3 and Figure 3c), ambient temperature (*p* = 0.312) (Table 3 and Figure 3d) average daily humidity (*p* = 0.155) or total daily rainfall (*p* = 0.408). For each cohort of cows, the average daily MeY was not related to average daily wind speed (*p* = 0.313) (Table 3 and Figure 3e), average daily temperature (*p* = 0.375) (Table 3 and Figure 3f), average daily humidity (*p* = 0.566) or total daily rainfall (*p* = 0.794).

## 4. Discussion

Daily MeP and MeY were independent of normal day-to-day variation in average daily wind speed, temperature, relative humidity and rainfall. Thus, we accept our hypothesis. Wind speed was negatively correlated with the concentrations of both SF_6_ and methane in background and breath samples. This is consistent with the observations of Ulyatt et al. [17] and expected. High wind speed may dilute the samples of breath gases and the on-cow samples of background gases with clean background gas, that is, background gas with low concentrations of SF_6_ and methane similar to those reported for global background gas [31]. Ulyatt et al. [17] reported the mean concentrations of SF_6_ and of methane, as well as CH_4_/SF_6_ ratio in breath samples, MeP and average daily wind speed from 15 sheep that grazed perennial ryegrass and white clover pasture on five consecutive days. They did not present any data on SF_6_ and methane concentrations in samples of background gas nor did they present any relationships between wind speed and the gas parameters. However, consistent with our results, Ulyatt et al. [17] reported that the day with the lowest wind speed (2.5 m/sec), was the day when breath samples had the greatest average concentration of SF_6_ (1736 ppt) and greatest concentration of methane (90.8 ppm) and relatively low calculated MeP (16.9 g/day). In contrast, on the day with the highest wind speed (7.8 m/sec), the average concentrations of SF_6_ and methane in breath samples as well as the calculated MeP were 1197 ppt, 78 ppm, and 19.1 g/day, respectively. However, Ulyatt et al. [17] did not measure DMI, so they could not report methane yield, nor could they make an inference as to whether any difference in MeP on days of different wind speed was due to an effect of wind speed on DMI or an effect of wind speed on the SF_6_ technique per se. We found that DMI was not affected by wind speed. Thus, since DMI was not affected by wind speed, and calculated MeY was not affected by wind speed, we can conclude that the SF_6_ technique per se is not affected by wind speed.

Wind direction has been suggested as one of the major factors potentially affecting methane measurements in grazing studies [4,32]. However, as we have found that wind speed during our measurements did not influence the modified SF_6_ technique, and as cows orient themselves in many directions at different times during the day, we also surmise that wind direction does not influence the technique.

The effects of ambient temperature on concentrations of SF_6_ and methane were different for background and breath samples. The concentrations of SF_6_ and methane in background gas samples were unrelated to ambient temperature, as expected. However, concentrations of SF_6_ and methane in the breath samples were negatively related to air temperature, which was contrary to expectations. Our conjecture was that the concentration of gases in breath samples would be positively related to air temperature since an increase in temperature should decrease the difference in density between air and breath, thereby reducing dispersion and increasing the concentration of breath gases in the sample. Our results suggest that mechanisms other than gas density affect breath sampling efficiency. As air temperature increases, the time cows spend eating decreases [33]. However, DMI was not affected by temperature, most likely since the cows had the opportunity to eat overnight when temperatures were lower. We speculate that a more likely effect on breath sampling efficiency is respiration rate, which increases as temperature increases [34,35]. As respiration rate increases, the velocity of gas expiration increases and therefore, gases may be breathed away from the sampling point which reduces the sampling efficiency, as observed in our results. We were not able to find any previous reports on the effect of temperature on the estimation of MeP and MeY using the SF_6_ technique, so additional research is necessary to test our speculation.

Relative humidity tended to have a negative effect (*p* = 0.083) on the concentration of SF_6_ and had a negative effect (*p* = 0.030) on methane in background samples, but no effect in breath samples. The reason for this is unclear. For breath samples, the absence of an effect of humidity on gas concentration adds further weight to the argument that differences in gas density do not explain the differences in gas concentrations in the collected samples.

Total daily rain was positively correlated with SF_6_ and methane in both background and breath samples. Since rain is independent of both DT and DH, we speculate that animal behavior may provide the explanation. Weather has been reported to influence a range of behaviors of cattle kept outdoors [27,36,37]. If animals huddle together during rainfall as a means of seeking shelter, then for any particular animal, the breath of that animal’s neighbors will increase the concentration of both SF_6_ and methane in that particular animal’s background sample. We also speculate that the huddling of animals during rain will somehow reduce the dispersion of breath and thereby enable increased sampling efficiency with commensurate increases in gas concentrations. 

The concentrations of SF_6_ and methane in the background samples from our experiment were substantially greater than the 9.5 ppt for SF_6_ [31] and 1.85 ppm for methane [38] that are the current global background concentrations for these gases. Our findings indicate that even though the cows in this investigation were kept outdoors for 23 h/day and were indoors for less than 1 h/day during milking, the background gas to which individual cows were exposed, was likely contaminated by the SF_6_ and methane from their herd mates. However, for these gases, our background concentrations which were measured in samples collected outdoors on the back of individual cows, were much lower than the 33.7 ± 6.4 ppt for SF_6_ and 10.0 ± 2.1 ppm for methane as reported for samples collected inside a barn [39]. Lassey [21] pointed out that the accuracy of the SF_6_ technique depends on having background concentrations of SF_6_ and methane that are much smaller than their breath counterparts. We also noticed considerable variation between individual cows. For example, one day during the experiment, an individual cow kept to herself while the remaining 39 cows in the cohort tended to huddle together (Figure 1b). The background SF_6_ concentration for this lone cow was 6.3 ppt but averaged 12.1 ± 1.95 ppt for the huddled cows. Similarly, the background methane concentration was 6.5 ppm for the lone cow and averaged 7.6 ± 1.02 ppm for the huddled cows. Our observations on individual cow behavior as exemplified in Figure 1b, lead us to surmise that an individual cow’s behavior, including proximity to herd-mates, may influence the background concentrations of SF_6_ and methane to which she is exposed. For this reason, we support the measurement of background gases for individual cows rather than the use of a single measure of background gases for all cows in a herd or the use of sentinel canisters placed up and down wind of experimental animals [40].

Our results may not be applicable to other techniques for estimating the MeP of animals kept outdoors. Both the micrometeorological [41] and the Greenfeed [42] methods have different mechanics to determine MeP so our results from the SF_6_ technique cannot be used to make inferences about how weather might affect those other methods.

We acknowledge that the ambient weather conditions in our experiment were relatively mild as the wind speeds ranged from 0 to 9.3 m/sec, temperature from 3.0 to 34.1 °C, relative humidity from 0.3 to 100% and rainfall from 0 to 20 mm/d. Further research is required to determine if weather conditions outside the range of those encountered in this experiment may impact on the measurement of MeP and MeY by the SF_6_ technique.

## 5. Conclusions

This research has shown that the modified SF_6_ technique can be used outdoors during a range of weather conditions without causing a significant effect on the measurement of MeP or MeY of dairy cows. Substantial between-cow and between-day differences in concentrations of SF_6_ and methane in both background samples and breath samples highlight the need for the correction of background gases for individual cows in the SF_6_ technique and for measurements to be made over multiple days. 

## Figures and Tables

**Table 1:** Composition of the diet. Units are (g/kg, DM) unless otherwise stated.

Item	2015	2016	2017	Average
Dry matter	863	874	870	869
Crude protein	190	168	173	177
Soluble protein (% of CP)	34.2	36.1	30.3	33.5
Acid detergent fiber	289	316	316	307
Neutral detergent fiber	357	380	371	369
Lignin	71	72	78	74
Non-fiber carbohydrate	338	348	358	348
Starch	92	110	124	109
Crude fat	21	18	21	20
Metabolizable energy (MJ/kg, DM)	10.1	9.8	10.0	10.0
Gross energy (MJ/kg, DM)	17.7	17.1	18.2	17.7
Ash	94	86	77	86
Calcium	11.7	10.4	12.0	11.4
Magnesium	3.1	3.1	3.2	3.1
Phosphorus	3.3	3.0	3.5	3.3
Potassium	25.8	25.1	16.4	22.4
Sodium	1.1	0.6	0.7	0.8
Iron (ppm)	212	179	257	216
Zinc (ppm)	77	70	63	70
Copper (ppm)	25	24	21	23
Manganese (ppm)	74	70	59	68
Sulfur	3.2	3.1	3.0	3.1
Chloride ion	6.9	5.9	4.2	5.7
DCAD (mEq/100 g, DM)	32	31	15	26

**Table 2:** Weather conditions during the 30 days (5 days for each of 6 cohorts) of the experiment when the SF_6_ technique was used to measure MeP.

Item.	Average	Standard Deviation	Minimum	Maximum
Average daily wind speed (m/sec)	1.7	0.69	0.5	3.5
Maximum daily wind speed (m/sec)	4.9	1.80	1.6	9.3
Minimum daily wind speed (m/sec)	0.1	0.25	0.0	1.3
Average daily temperature (°C)	16.5	4.19	9.0	23.8
Maximum daily temperature (°C)	23.0	6.33	14.0	34.1
Minimum daily temperature (°C)	10.9	3.37	3.0	16.9
Average daily relative humidity (%)	79.7	11.3	51.4	97.9
Maximum daily relative humidity (%)	99.4	1.68	93.1	100.0
Minimum daily relative humidity (%)	54.0	17.0	15.2	84.8
Total daily rainfall (mm)	3.0	4.99	0.0	20.0

**Table 3:** Influence of mean daily wind speed (DW, m/sec), mean daily air temperature (DT, °C), mean daily relative humidity (DH, %) and daily total rain (DR, mm) on concentrations in background gases of SF_6_ (BGSF6, ppt) and methane (BGCH4, ppm) and concentrations in breath of SF_6_ (CBSF6, ppt) and of methane (CBCH4, ppm), as well as dry matter intake (DMI, kg/day), calculated methane production (MeP, g/day) and methane yield (MeY, g/kg DMI).

Equation	R^2^	*p* Values			
		Wind	Air Temp	Humidity	Rainfall
BGSF6 = 25.6 ± 4.79 − 1.9 ± 0.40 DW + 0.020 ± 0.091 DT − 0.072 ± 0.040 DH + 0.10 ± 0.046 DR	0.29	0.001	0.825	0.083	0.040
BGCH4 = 23.3 ± 5.21 − 2.3 ± 0.44 DW − 0.04 ± 0.099 DT − 0.10 ± 0.043 DH + 0.16 ± 0.051 DR	0.21	0.001	0.667	0.030	0.004
CBSF6 = 109 ± 19.1 − 5.2 ± 1.57 DW − 1.5 ± 0.36 DT − 0.13 ± 0.16 DH + 0.63 ± 0.18 DR	0.22	0.003	0.001	0.419	0.002
CBCH4 = 97.9 ± 19.87 − 5.3 ± 1.67 DW − 1.39 ± 0.38 DT − 0.14 ± 0.16 DH + 0.59 ± 0.19 DR	0.29	0.004	0.001	0.399	0.006
DMI = 24.3 ± 4.25 + 0.29 ± 0.35 DW − 0.005 ± 0.080 DT + 0.008 ± 0.035 DH − 0.014 ± 0.040 DR	0.51	0.410	0.954	0.830	0.729
MeP = 750 ± 141.3 − 26.6 ± 14.2 DW − 2.6 ± 2.51 DT − 1.8 ± 1.22 DH − 1.3 ± 1.59 DR	0.14	0.072	0.312	0.155	0.408
MeY = 25.8 ± 6.26 − 0.56 ± 0.544 DW − 0.11 ± 0.119 DT − 0.031 ± 0.053 DH − 0.016 ± 0.062 DR	0.03	0.313	0.375	0.566	0.794

## Data Availability

Data are available upon request to the corresponding author.
